# Glutamate Dehydrogenase Is Required by *Mycobacterium bovis* BCG for Resistance to Cellular Stress

**DOI:** 10.1371/journal.pone.0147706

**Published:** 2016-01-29

**Authors:** James L. Gallant, Albertus J. Viljoen, Paul D. van Helden, Ian J. F. Wiid

**Affiliations:** 1 DST-NRF Centre of Excellence for Biomedical Tuberculosis Research, SA MRC Centre for Tuberculosis Research, Division of Molecular Biology and Human Genetics, Faculty of Medicine and Health Sciences, Stellenbosch University, Tygerberg, Cape Town, South Africa; 2 Centre National de la Recherche Scientifique FRE 3689, Centre d’études d’agents Pathogènes et Biotechnologies pour la Santé, Université de Montpellier, 1919 route de Mende, Montpellier, France; University of Padova, Medical School, ITALY

## Abstract

We recently reported on our success to generate deletion mutants of the genes encoding glutamate dehydrogenase (GDH) and glutamine oxoglutarate aminotransferase (GOGAT) in *M*. *bovis BCG*, despite their *in vitro* essentiality in *M*. *tuberculosis*. We could use these mutants to delineate the roles of GDH and GOGAT in mycobacterial nitrogen metabolism by using *M*. *bovis* BCG as a model for *M*. *tuberculosis* specifically. Here, we extended our investigation towards the involvement of GDH and GOGAT in other aspects of *M*. *bovis* BCG physiology, including the use of glutamate as a carbon source and resistance to known phagosomal stresses, as well as in survival inside macrophages. We find that *gdh* is indispensable for the utilization of glutamate as a major carbon source, in low pH environments and when challenged with nitric oxide. On the other hand, the *gltBD* mutant had increased viability under low pH conditions and was unaffected by a challenge with nitric oxide. Strikingly, GDH was required to sustain *M*. *bovis* BCG during infection of both murine RAW 264.7 and bone-marrow derived and macrophages, while GOGAT was not. We conclude that the catabolism of glutamate in slow growing mycobacteria may be a crucial function during infection of macrophage cells and demonstrate a novel requirement for *M*. *bovis* BCG GDH in the protection against acidic and nitrosative stress. These results provide strong clues on the role of GDH in intracellular survival of *M*. *tuberculosis*, in which the essentiality of the *gdh* gene complicates knock out studies making the study of the role of this enzyme in pathogenesis difficult.

## Introduction

Rapidly increasing numbers globally of tuberculosis (TB) patients infected with *Mycobacterium tuberculosis* strains resistant to many TB drugs underscores an urgent need for the development of new TB chemotherapies acting by mechanisms that are distinct from the current anti-TB drugs [1]. The central carbon and nitrogen metabolism of *M*. *tuberculosis* is composed of unique pathways that often are crucial to the survival of this organism and therefore represents a reservoir of enzymes that may be exploited as targets for novel anti-TB chemotherapy development [2,3].

**Fig 1:**
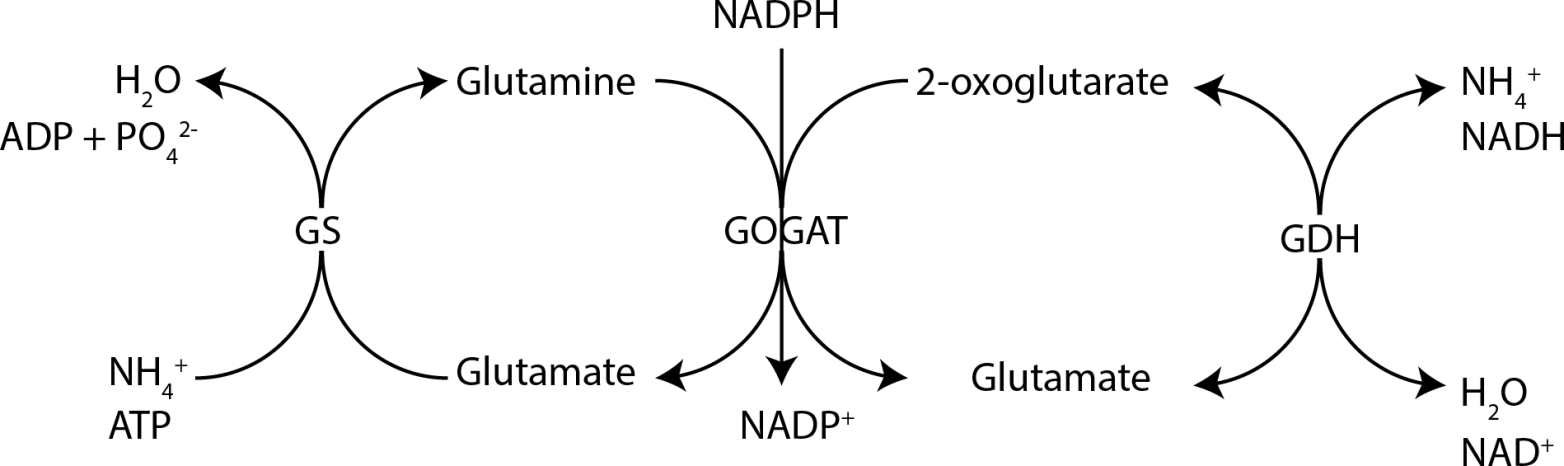
Schematic illustration depicting central nitrogen metabolism in the mycobacterium tuberculosis complex.

In particular the involvement of protein kinase G (PknG) and glycogen accumulation factor A (GarA), has been implicated in the regulation of enzymes that are effectors of central carbon metabolism (CCM) and central nitrogen metabolism (CNM) [4–8]. It was found that binding of GarA to the ketoglutarate dehydrogenase complex (KDH), glutamate dehydrogenase (GDH) and glutamine oxoglutarate aminotransferase (GOGAT), inhibits KDH and GDH activity, while it stimulated GOGAT activity [6,7]. Activated GOGAT promotes glutamate accumulation and subsequently removes 2-oxoglutarate which results in cataplerosis of the TCA cycle and nett glutamate accumulation (Fig 1) [9]. Phosphorylation of GarA by PknG abrogated the interaction between GarA and KDH/GDH/GOGAT resulting in nett glutamate degradation and accumulation of 2-oxoglutarate resulting in anaplerosis of TCA cycle intermediates [6,7,9]. These results implicated regulation of glutamate metabolism as an important factor of *M*. *tuberculosis* physiology. Strikingly the genes encoding GDH and GOGAT were found to be essential for *in vitro* growth of *M*. *tuberculosis* further emphasising a requirement for homeostatic regulation of glutamate by this pathogen [10–12]. However, a mutant of the gene encoding PknG could be obtained in *M*. *tuberculosis* allowing the delineation of the role of this virulence factor in pathogenesis [4]. An *M*. *smegmatis* conditional *garA* mutant was also recently generated to study the role of GarA in mycobacterial physiology more clearly, but its essentiality in *M*. *tuberculosis* has complicated efforts to study its involvement in pathogenesis [8]. Similarly the essentiality of *gdh* and *gltBD* has probably hindered attempts to more clearly define their specific contributions to pathogenicity. However, we recently reported on our success to generate mutants of these genes in *M*. *bovis BCG*, which is highly similar to *M*. *tuberculosis* at a genetic level [13]. With the use of these mutants we could delineate the roles of these two enzymes in the nitrogen metabolism of *M*. *bovis BCG*, by showing that GOGAT was essential for growth on ammonium as a sole nitrogen source, while GDH was required for optimal growth on glutamate as a sole nitrogen source and when high levels of asparagine was present in the growth medium [13]

Here we have used our *M*. *bovis* BCG *gdh* and *gltBD* mutants to investigate more clearly their importance in intracellular survival by using *in vitro* conditions that simulate some of the stresses employed by macrophages to kill infecting mycobacteria. We demonstrate an absolute requirement for *gdh* under limiting carbon conditions when glutamate was a major carbon source. We also demonstrate a protective role for *gdh* under nitrosative and acidic stress conditions. Surprisingly, *gltBD* was dispensable for survival under all of the aforementioned conditions. Finally, we use two murine macrophage systems to demonstrate that *gdh* is required for optimal survival of *M*. *bovis* BCG inside macrophages. Ours is the first report to implicate *gdh* in the intracellular survival of a mycobacterium.

Our results indicate that the catabolism of glutamate is an important process in *M*. *bovis* BCG which is primarily catalysed by the *gdh* protein product. While the enzyme product of the *gltBD* operon, glutamine oxoglutarate aminotransferase (GOGAT) has received some attention as a possible anti-TB drug target due to inhibition of *M*. *tuberculosis* by azaserine (an inhibitor of GOGAT) *in vitro* [14,15], our findings suggest that GDH may be as important if not more to the survival of *M*. *tuberculosis* inside host macrophage cells. Notably, the *M*. *tuberculosis* GDH is structurally and functionally different from the GDH found in humans as well as in the human intestinal flora, which may make this enzyme a potential specific target for anti-TB drug intervention [15].

## Materials and Methods

### Cultivation of bacteria

All bacterial strains used are listed in S2 Table. *M*. *bovis* BCG was cultured without agitation in 7H9 (BD scientific, USA) supplemented with 0.05% Tween 80, 0.2% glycerol, 5 g/L albumin, 2 g/L glucose and 1.5 mg/L catalase in 25 cm^2^ cell culture flasks (Nunc, Denmark). The *ΔgltBD* mutant which had a growth deficiency in standard 7H9 was maintained in 7H9 supplemented with 20 mM L-glutamate. In certain cases (indicated in the text), bacterial strains were cultivated in 7H9 that was not supplemented with Tween 80, glycerol, albumin, glucose or catalase. Genetically complemented strains were maintained under selection with gentamycin at 2.5 μg/ml.

### Nitric oxide challenge

All strains were challenged with diethelenetriamine/nitric oxide adduct (DETE/NO) (Sigma-Aldrich, USA). DETE/NO was stored as at -20°C as a lyophilised powder prior to use. The nitric oxide donor was prepared by solubilising DETE/NO in ultra-pure water to a final concentration of 5 M and immediately used as required.

*M*. *bovis* BCG, *Δgdh*, *Δgdh*: complement, *ΔgltBD* and *ΔgltBD* complement were cultivated as explained in the cultivation of bacteria section albeit slightly modified. OADC was replaced with ADN (Bovine serum albumin fraction V, 50g/L; D-dextrose, 20 g/L; sodium chloride, 8.5 g/L) as an alternative enrichment to avoid catalase. Upon reaching an OD_600_ of 0.8–1.0 all cultures were diluted to an OD_600_ of 0.0005 in 5 ml 7H9 supplemented with ADN and tween followed by challenge with 500 μM DETE/NO for a total period of 48 hours. Cells were plated on Middlebrook 7H11 agar (BD scientific, USA) supplemented with 0.2% glycerol and OADC at various time points (0h, 6h, 12h, 24h, 36h, 48h) and cell viability was determined by CFU.

### Culturing and infection of RAW 264.7 macrophages

RAW 264.7 ATCC^®^ TIC-71^™^ macrophages (ATCC, USA) were maintained in DMEM (Lonza, USA) at 37°C under 5% CO_2_ until confluent. Viable RAW 264.7 ATCC TIB-71 cells were quantified by staining with trypan blue and counted using a haemocytometer. Macrophages in DMEM supplemented with 10% FBS and 10 μg/ml LPS (Sigma Aldrich, USA) (D10) were seeded at 5 x 10^5^ cells/well in 24 well plates (Nunc, Germany) and incubated at 37°C under 5% CO_2_ overnight to allow the cells to adhere. Bacteria cultured to an OD_600_ of 0.8–1.0 in standard 7H9 without agitation at 37°C were pelleted and re-suspended in equal volumes of D10, which was then adjusted to an OD_600_ of 1.0 (corresponding to approximately 2.5 × 10^7^ CFU/100 μl). Bacteria were added directly to RAW 264.7 macrophage monolayers to obtain a multiplicity of infection (MOI) of 5:1 bacteria to macrophage and incubated at 37°C under 5% CO_2_ for 3 hours. The culture medium was subsequently removed and replaced with fresh D10 medium supplemented with penicillin/streptomycin (penstrep) antibiotic cocktail (1:100) and incubated for an additional hour at 37°C under 5% CO_2._ Monolayers were subsequently washed with PBS and supplemented with fresh D10. Infected RAW 264.7 macrophages were lysed by the addition of ultra-pure sterile water and scraping. Lysates were serially diluted prior to plating on 7H11 agar (BD scientific, USA). Enumerations of CFU were performed after approximately 2 weeks of culturing at 37°C when colonies started to appear.

### Culturing and infection of murine bone-marrow derived macrophages (BMDM)

Culturing and infection of BMDM macrophages was performed as previously described with some modifications [16]. Tibia and femur bones from 6–10 week old female C57/BL6 mice bred and housed under specific pathogen-free conditions were obtained from the Stellenbosch University animal facility. The extraction of bones from the aforementioned mice was carried out in strict accordance with the recommendations as reflected in the South African National Standards 10386: 2008.

The protocol, SU-ACUD14-00041, was approved by the research ethics committee: animal care and use of the University of Stellenbosch. Mice were euthanized prior to the extraction of tibia and femur bones by cervical dislocation. The bones were dissected and the marrow flushed out and dispersed with 2.5 ml of RPMI 1640 medium with L-glutamine and sodium bicarbonate (Sigma, USA) and a 25-GA syringe needle. The bone marrow cell suspension was adjusted to 2 × 10^5^ cells/ml in RPMI-LCSF20 medium (consisted of RPMI 1640 medium with L glutamine and sodium bicarbonate, 10% fetal bovine serum (Biochrom, UK) and 20% supernatant from L929 cells. The cells were plated in 10 ml volumes on 90 mm diameter Petri dishes (Greiner, USA) and incubated at 37°C with 5% CO_2_ for 7 days. Cells were fed with 10 ml RPMI-LCSF after 4 days. Non-adherent cells were removed in two washes with PBS (Lonza, USA) before adherent cells were released with 10 ml ice cold PBS. Released macrophages were adjusted to 2 × 10^5^ cells/ml in RPMI-LCSF10 (as described before but with 10% supernatant from L929 cells). Cells were seeded in 24 well plates (1 ml/well) and allowed to adhere for 24 hours. Bacteria grown to early exponential growth phase (OD_600_ = 0.5–0.8) were washed once with PBS (Lonza, USA). Bacterial aggregates were removed by low speed centrifugation (200 × g for 10 minutes) and the resulting supernatant passed through a 5 μm surfactant free cellulose acetate syringe filter (Sartorius, Germany). Bacterial suspensions were prepared in RPMI-LCSF10 to yield an MOI of 1:1. At this point the macrophage culture medium was replaced with 0.5 ml bacterial suspension. Infection of macrophages lasted for 4h before they were washed four times with pre-warmed PBS to eliminate non-phagocytosed bacteria and re-fed with RPMI-LCSF10. The medium covering each monolayer was replaced every 48h. Infected macrophages were lysed with 0.1% Triton X-100 solution (Sigma, Germany). Serial dilutions of the lysates were prepared in 7H9 and spread on 7H11 agar supplemented with OADC (BD scientific, USA). Bacterial colonies became visible and were enumerated after approximately 2 weeks of incubation at 37°C.

### Statistical analysis

Statistical analyses were carried out with the statistics software GraphPad Prism 5 version 5.01 (GraphPad Software). All statistical tests performed are indicated in figure legends. Probabilities of < 0.05 were considered significant.

## Results

### *gdh* is required for the utilization of glutamate as a major carbon source

**Fig 2:**
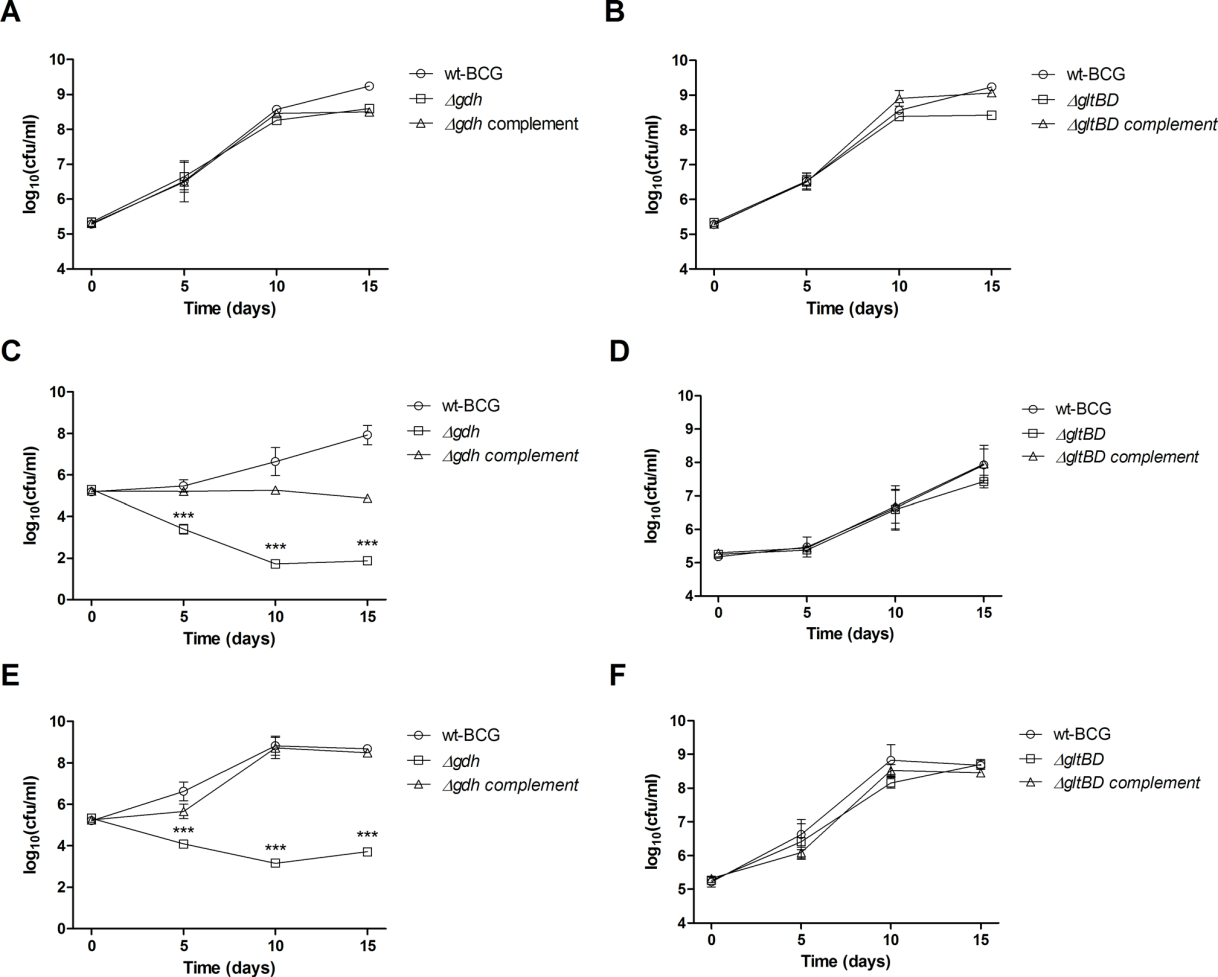
The effect of glutamate as a major carbon source on survival of *M*. *bovis* BCG wild type, mutant (*Δgdh* and *ΔgltBD*) and complemented strains. Exponential phase cultures were diluted to an OD_600_ of 0.0005 into (A & B) 7H9 supplemented with glycerol, dextrose and Tween 80, (C & D) 7H9 without glycerol, dextrose, or Tween 80 (replaced with tyloxapol) or (E & F) 7H9 without glycerol, dextrose, or Tween80 and supplemented with cholesterol (0.25 mM). Symbols and error bars are means and standard errors calculated from triplicate plating obtained from two independent experiments. Data was analysed by a regular two-way ANOVA test with Bonferroni post-testing to compare mean CFU/ml of mutant (*Δgdh* and *ΔgltBD*) and wild type strains. * p < 0.05, ** p < 0.01, *** p < 0.001.

We previously reported GDH’s importance for optimal growth of *M*. *bovis BCG* when glutamate was the sole nitrogen source and when cultured in the presence of excessive asparagine levels [13]. Since there is some evidence that glutamate may also be utilized as a carbon source by *M*. *tuberculosis* [17,18], we analysed the growth of *M*. *bovis BCG* in 7H9 medium which was not supplemented with albumin, dextrose or glycerol and in which the detergent was tyloxapol as opposed to tween 80. This medium, which contains glutamate as the major carbon source at 3.4 mM in addition to the carbon sources citrate (0.5 mM), pyridoxine (2.9 μM) and biotin (4.1 μM), could support growth of *M*. *bovis* BCG (Fig 2C), although the replication rate was lower than when the medium was supplemented with albumin, dextrose, glycerol and Tween 80 (Fig 2A). Strikingly the *Δgdh* mutant became non-viable in the medium containing glutamate as major carbon source with an approximately 3 log_10_ (CFU/ml) reduction in viable cell counts after 10 days of culture (Fig 2C). Notably this phenotype of the *Δgdh* mutant is much more severe than the phenotype we previously reported for the same mutant when it was cultured in medium containing glutamate as the sole nitrogen source, in which case replication rate was merely decreased and cultures reached a lower maximum density compared to wild type [13]. Also in contrast to earlier observations in medium containing glutamate as sole nitrogen source, the decrease in viability of the *Δgdh* mutant in medium containing glutamate as major carbon source was not completely reversed by re-introduction of an intact copy of *gdh* as the complemented strain remained static over the course of the experiment. This poor complementation corresponded with low specific GDH activity levels in crude lysates as well as lower mRNA expression of *gdh* in the genetically complemented strain compared to the wild type strain (S1 Table). It was shown that *M*. *tuberculosis* uses cholesterol as a major carbon source during infection of mice [19]. Therefore, we tested whether the addition of cholesterol to culture medium containing glutamate as major carbon source would chemically complement for the loss in viability suffered by the *Δgdh* mutant. Unexpectedly the *Δgdh* mutant continued to become non-viable in the medium containing cholesterol in addition to the major carbon source glutamate (Fig 2E), while the *ΔgltBD* mutant exhibited no apparent growth defects under the conditions tested (Fig 2B, 2D & 2E). However, addition of cholesterol improved growth of the *Δgdh* complemented strain to wild type levels (Fig 2E). Collectively these results indicate that GDH is indispensable for growth of *M*. *bovis* BCG under conditions when nutrients are limiting and some of the only available sources of both nitrogen and carbon is glutamate and related amino acids, such as glutamine; asparagine and aspartate that are easily converted to glutamate.

### Inactivation of *gdh* increases sensitivity to low pH stress

**Fig 3:**
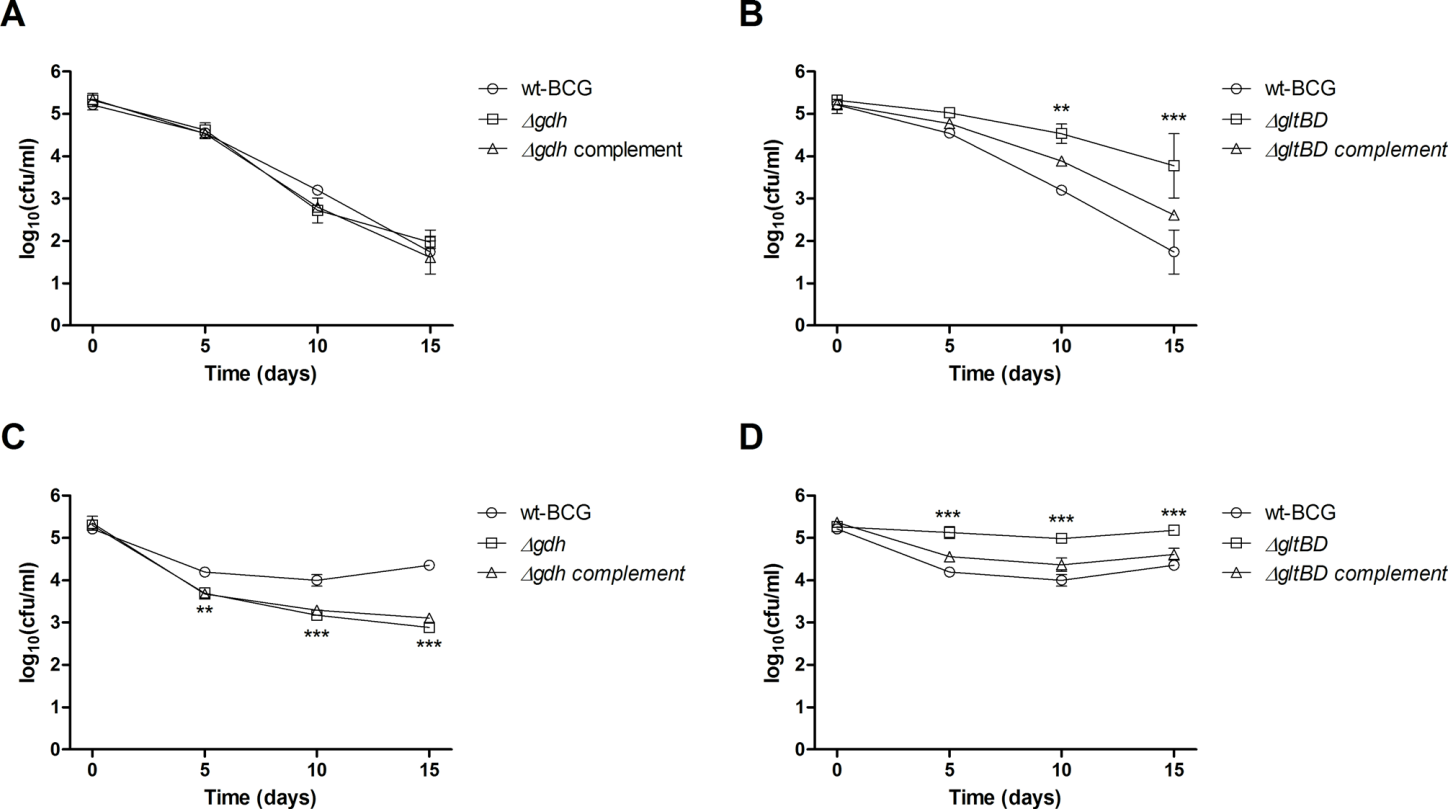
The effect of pH on survival of *M*. *bovis* BCG wild type, mutant (*Δgdh* and *ΔgltBD*) and complemented strains. Exponential phase cultures were diluted to an OD_600_ of 0.0005 into 7H9 of which the pH was adjusted to 4.5 (A & B) or 5.5 (C & D). Symbols and error bars are means and standard errors calculated from triplicate plating obtained from two independent experiments. Data was analysed by a regular two-way ANOVA test with Bonferroni post-testing to compare mean CFU/ml of mutant (*Δgdh* and *ΔgltBD*) and wild type strains. * p < 0.05, ** p < 0.01, *** p < 0.001.

It is well documented that macrophages acidify their intracellular environment in response to invading pathogens [20,21]. It has been proposed that *M*. *tuberculosis* is able to circumvent macrophage acidification through release of ammonia, which can act as an alkalising agent in an acidic environment by sequestering hydrogen ions to produce ammonium [22,23]. Since GDH converts glutamate to ammonia, we hypothesised that GDH might also play a role in offering protection against acidic stress. While replication was observed for both the *Δgdh* and *ΔgltBD* mutants as well as the wild type strain after about 1 week of incubation in 7H9 medium of which the pH was adjusted to 4.5 with HCl, CFU enumerations for an additional week showed that replication of only the *Δgdh* mutant was inhibited by the acidic medium (Fig 3A). This inhibition of bacterial division was less pronounced at pH 5.5, but the replication rate of the *Δgdh* mutant was still reduced in comparison to the wild type strain and *Δgdh* complemented strain (Fig 3C). Strikingly, growth of the *ΔgltBD* mutant was unaffected by the low pH conditions investigated in the current study (Fig 3B and 3D). In contrast, deletion of *gtlBD* offered marginal protection against acidic stress at pH 4.5 (Fig 3B) and pH 5.5 (Fig 3D) and in both instances re-introduction of an intact copy of the *gltBD* operon restored sensitivity to wild type levels. These results indicate that degradation of glutamate through the deaminating activity of GDH offers some degree of protection against an acidic environment, probably through the release of buffering ammonia molecules into the milieu. However, our observation that a deficiency in glutamate production through GOGAT activity also confers some tolerance to acidic stress, may indicate that resistance to acidic stress may be directly correlated with intracytosolic glutamate levels, which is likely to be decreased in the *ΔgltBD* mutant and increased in the *Δgdh* mutant.

### Inactivation of *gdh* increases sensitivity to nitrosative stress

**Fig 4:**
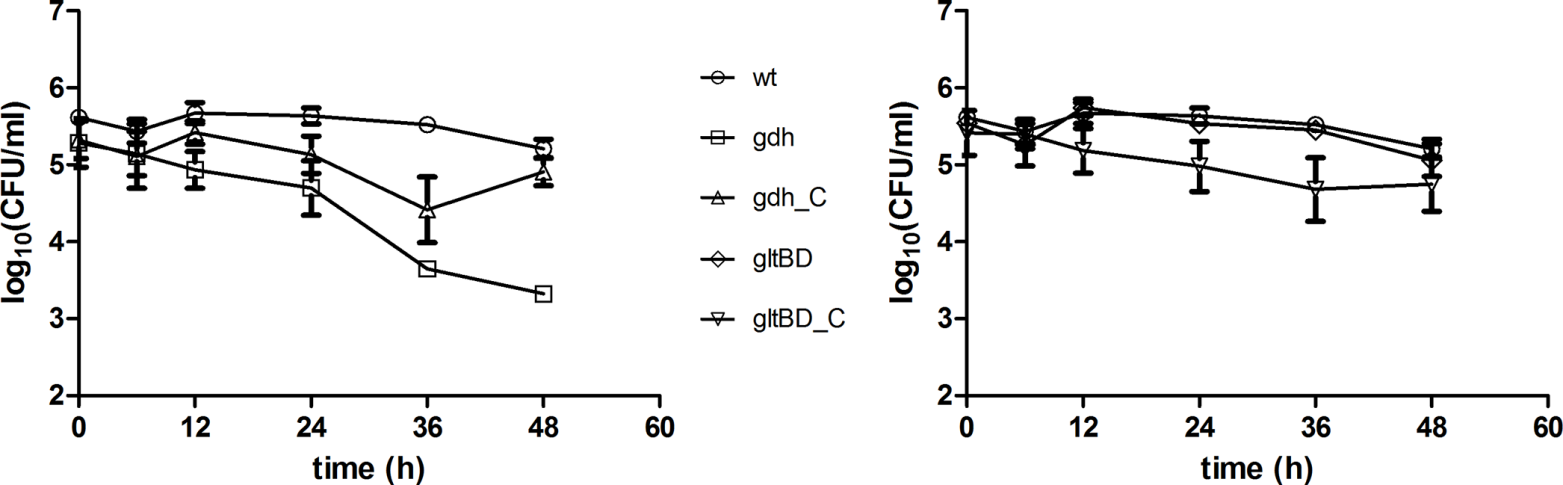
The effect of diethylenetriamine/nitric oxide adduct (DETE/NO) exposure on survival of *M*. *bovis* BCG wild type, mutant (*Δgdh* and *ΔgltBD*) and complemented strains. Exponential phase cultures of wild type, *Δgdh* and *Δgdh* complement (A) or *ΔgltBD* and *ΔgltBD* complement (B) were diluted to an OD_600_ of 0.0005 into 7H9 (without catalase), which was supplemented with 500 μM of diethylenetriamine/nitric oxide adduct (DETE-NO) (Sigma-Aldrich, USA) and cultured for 48 hours at 37°C without agitation. Symbols and error bars are means and standard errors calculated from three independent experiments. Data was analysed by a regular two-way ANOVA test with Bonferroni post-testing to compare mean CFU/ml of mutant (*Δgdh* and *ΔgltBD*) and wild type strains. * p < 0.05, ** p < 0.01, *** p < 0.001.

Nitric oxide (NO) is an essential component of the innate immune defence against invading pathogens and is known to play an important role in the defence of murine macrophages against *M*. *tuberculosis* infection [24]. To test the effect of nitrosative stress on *M*. *bovis* BCG and the involvement of *gdh* and *gltBD*, the wild type, mutant and complemented strains were challenged for 48 hours with 500 μM of diethelenetriamine/nitric oxide adduct (DETE/NO) in 7H9 lacking catalase. Under this *in vitro* nitrosative condition, division of the wild type strain was inhibited over the 48 hour period, but bacteria remained viable as no significant reduction in CFU was observed over this period (Fig 4A). However the *Δgdh* mutant exhibited a decline in viability, with a 2 log difference at 48 hours compared to wild type and complemented strains (Fig 4A). Interestingly, when the *gdh* mutant was cultivated with excess ammonium sulphate (20 mM and 30 mM) there was no decline in cell viability observed at the 48 hour time point post challenge (S1 Fig). A significant decline in viability was not observed for the *ΔgltBD* mutant (Fig 4B). These results indicate that the degradation of glutamate to produce 2-oxoglutarate and ammonia offers resistance to the nitrosative effect of NO.

### *gdh* is required for optimal growth in murine macrophages

**Fig 5:**
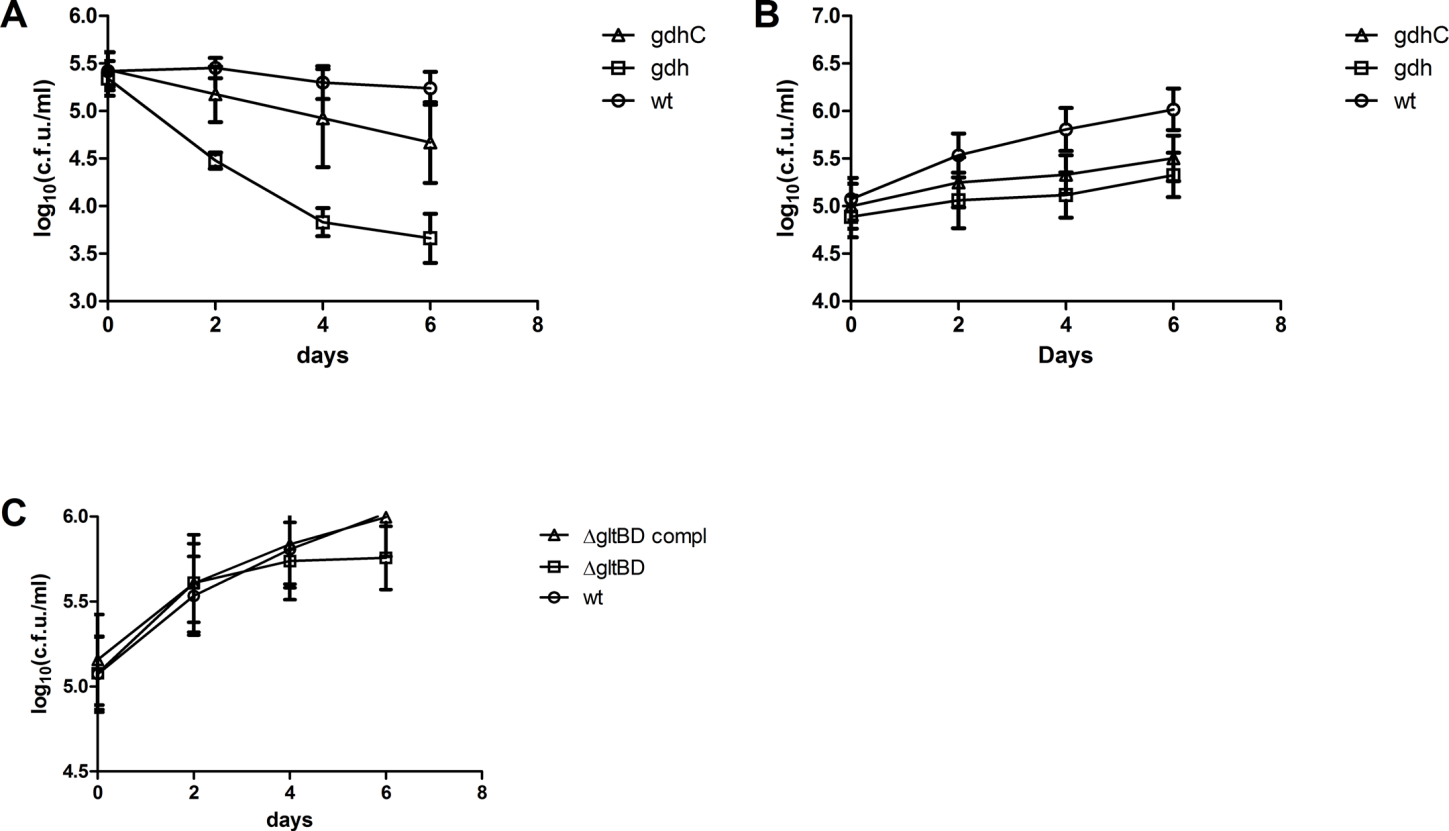
Survival of *M*. *bovis* BCG wild type, *Δgdh* mutant and *Δgdh* complemented strains in macrophages. RAW 264.7 macrophages (A) or BMDM (B) were infected with *M*. *bovis* BCG wild type, *Δgdh* mutant and *Δgdh* complemented strains or (C) the *ΔgltBD* mutant and complemented strains and the survival of the bacteria was determined by measuring CFU obtained from macrophage lysates. Means and standard errors were calculated from three independent experiments. For the BMDM infections a different mouse was used in each experiment. Data was analysed by repeated measures two-way ANOVA with Bonferroni post-testing to compare mean CFU/ml of *Δgdh* mutant and wild type strains. * p < 0.05, ** p < 0.01, *** p < 0.001.

In order to determine whether the contribution of *gdh* in *M*. *bovis BCG* resistance to acidic and nitrosative stress *in vitro* is actually relevant to bacterial survival inside host macrophages, we used murine macrophages as a cellular model of mycobacterial infection. As a start we studied the survival of the wild type *Δgdh* mutant and complemented strains in RAW 264.7 macrophages stimulated with lipopolysaccharides (LPS) from *Escherichia coli*, which is a macrophage cell line and represented a simple model system. While *M*. *Bovis* BCG maintained an equivalent bacterial load for the duration of infection in RAW 264.7 macrophages, *Δgdh* became non-viable with a nearly 2 log reduction in CFU after 6 days of infection (Fig 5A). Complementation improved viability of the mutant significantly, but not to the level seen for the wild type strain (Fig 5A). To corroborate our observation that the survival of the *Δgdh* mutant is reduced in macrophages, we repeated the infection experiment using the *ex vivo* murine bone-marrow derived macrophage (BMDM) model and including the *ΔgltBD* mutant and complement in the assay. In contrast to our earlier finding in RAW 264.7 macrophages, wild type *M*. *bovis* BCG growth was not suppressed in BMDM, but the replication rate of the *Δgdh* mutant was significantly impaired in comparison to the wild type strain (Fig 5B). Complementation of the *Δgdh* mutant did not restore the wild type replication rate (Fig 5B), probably due to poor expression of GDH (S1 Table). In contrast, the replication rate of the ΔgltBD mutant was not significantly different from the wild type strain in BMDM (Fig 5C). These results strongly implicate GDH in playing a substantial role in protecting infecting tubercle bacilli against host macrophage defence strategies.

## Discussion

We previously reported on our success to inactivate both *gdh*, which encodes the primary enzyme of glutamate catabolysis, and *gltBD*, which encodes the primary enzyme of glutamate synthesis, in *M*. *bovis BCG*, despite their *in vitro* essentiality in closely related *M*. *tuberculosis* [13]. Constructing these mutants allowed us to study the roles of these genes and their protein products in nitrogen metabolism in *M*. *bovis* BCG as a model slow growing mycobacterium. We showed that *gdh* is required for the utilisation of glutamate as a sole nitrogen source and when the amino acids asparagine and aspartate are present in excess concentrations [13]. On the other hand we found that *M*. *bovis* BCG became glutamate auxotrophic when *gltBD* was inactivated [13]. This glutamate auxotrophy could however be rescued by supplementation with amino acids such as aspartate, glutamine and asparagine [13].

In addition to a role as a precursor or nitrogen donor in the biosynthesis of a range of nitrogenous molecules [25,26], glutamate may be involved to a great extent in other functions that may be critical to the intracellular lifestyle and pathogenesis caused by slow growing mycobacterial pathogens such as *M*. *tuberculosis*. These include a source of carbon in its carbon backbone structure which may be freed as a 2-oxoglutarate molecule (a TCA cycle intermediate), a function in acid resistance [27,28], osmoprotection [29] and as a compatible solute [30]. Therefore, we extended our study of glutamate metabolism here by using the same mutants and *in vitro* conditions that mimic some of the bactericidal mechanisms employed by macrophage cells as well as two macrophage infection models to further delineate the role of glutamate in the pathogenicity of slow growing mycobacteria. We show that *gdh* is indispensable for the utilisation of glutamate as a major carbon source even in the presence of cholesterol, a neutral lipid which was previously shown to constitute a major source of carbon for *M*. *tuberculosis* in a chronic infection mouse model [19,31]. Mycobacterial central metabolism is different when cultured in medium containing in addition to asparagine, cholesterol or glycerol as major carbon sources, with central metabolites such as succinate, fumarate and malate accumulating only in the medium containing cholesterol [32]. Interestingly, these methylcitrate cycle intermediates are also downstream products of glutamate catabolism through the release of 2-oxoglutarate. It could be speculated that the poor growth of the *Δgdh* mutant in medium containing both cholesterol and glutamate as carbon sources is a result of deregulation associated with 2-oxoglutarate metabolism, which is not ameliorated by supplementation with cholesterol, however a deeper investigation is warranted to understand the interplay between glutamate and cholesterol catabolism. We also show that *gdh* is required for survival in a low pH environment as well as under nitrosative stress, two defence mechanisms employed by macrophages to destroy phagocytosed microbes [20,21]. Under none of the tested conditions could we show a critical involvement for *gltBD*. Finally, we show that *gdh* is required for optimal survival of *M*. *bovis* BCG in murine macrophages.

Compellingly, our results are in agreement with recent studies into the uptake and metabolism of the amino acids glutamine, asparagine and aspartate, which are easily converted to glutamate, and their involvement in survival and pathogenicity of *M*. *tuberculosis*. Especially the highly expressed essential ammonium assimilating enzyme glutamine synthetase (GS), has garnered a lot of attention for its involvement in *M*. *tuberculosis* virulence [22,33–37]. However, this enzyme is mostly important under conditions when amino acids are not available as sources of nitrogen and only ammonium is available for the *de novo* synthesis of glutamine [22,33,38]. It was more recently found that a putative asparagine/aspartate transporter (AnsP1) is able to transport aspartate across the cell envelope and deletion of *ansP1* resulted in impaired virulence of *M*. *tuberculosis* in a mouse model, clearly demonstrating that the assimilation of aspartate by *M*. *tuberculosis* is required for virulence [39]. The same group inactivated *ansP2*, which encodes another permease transporting asparagine across the cell envelope, but found this gene to be non-essential to virulence in mice [40]. However, when they inactivated *ansA*, which encodes an asparaginase, which readily converts asparagine to aspartate and ammonia a growth defect was observed in mice [40]. Interestingly, AnsA is secreted and it was speculated by the authors that the conversion of asparagine to aspartate is necessary for virulence due to the release of ammonia to the *M*. *tuberculosis* extracellular environment [40]. Ammonia may alkalise the phagosome and circumvent acidification and maturation, two crucial processes of macrophage defence against intracellular infection [23]. Through this mechanism, *M*. *tuberculosis* can initiate an efficient response against the host defences while simultaneously gaining nutrition in the form of nitrogen and carbon through the assimilation of aspartate. In *M*. *tuberculosis*, aspartate could be transaminated by at least three putative aspartate aminotransferases (AspB, AspC and Rv3722c) to produce glutamate, which in turn could be deaminated by GDH to produce free ammonia and 2-oxoglutarate.

Our observation that *gdh* is also an important factor in protection against nitrosative stress was unanticipated. It has been found that high levels of intracellular ammonia may induce the expression of enzymes involved in protection against oxidative stress [40,41–43]. In addition, it was more recently found that 2-hydroxy-3-oxoadipate synthase (HOAS) of the KDH complex in *M*. *tuberculosis* forms part of a four component peroxidase system which uses 2-oxoglutarate as an electron donor in a reductase reaction [44], which might also implicate 2-oxoglutarate in protection against nitrosative stress as a product of glutamate degradation through GDH activity.

**Fig 6:**
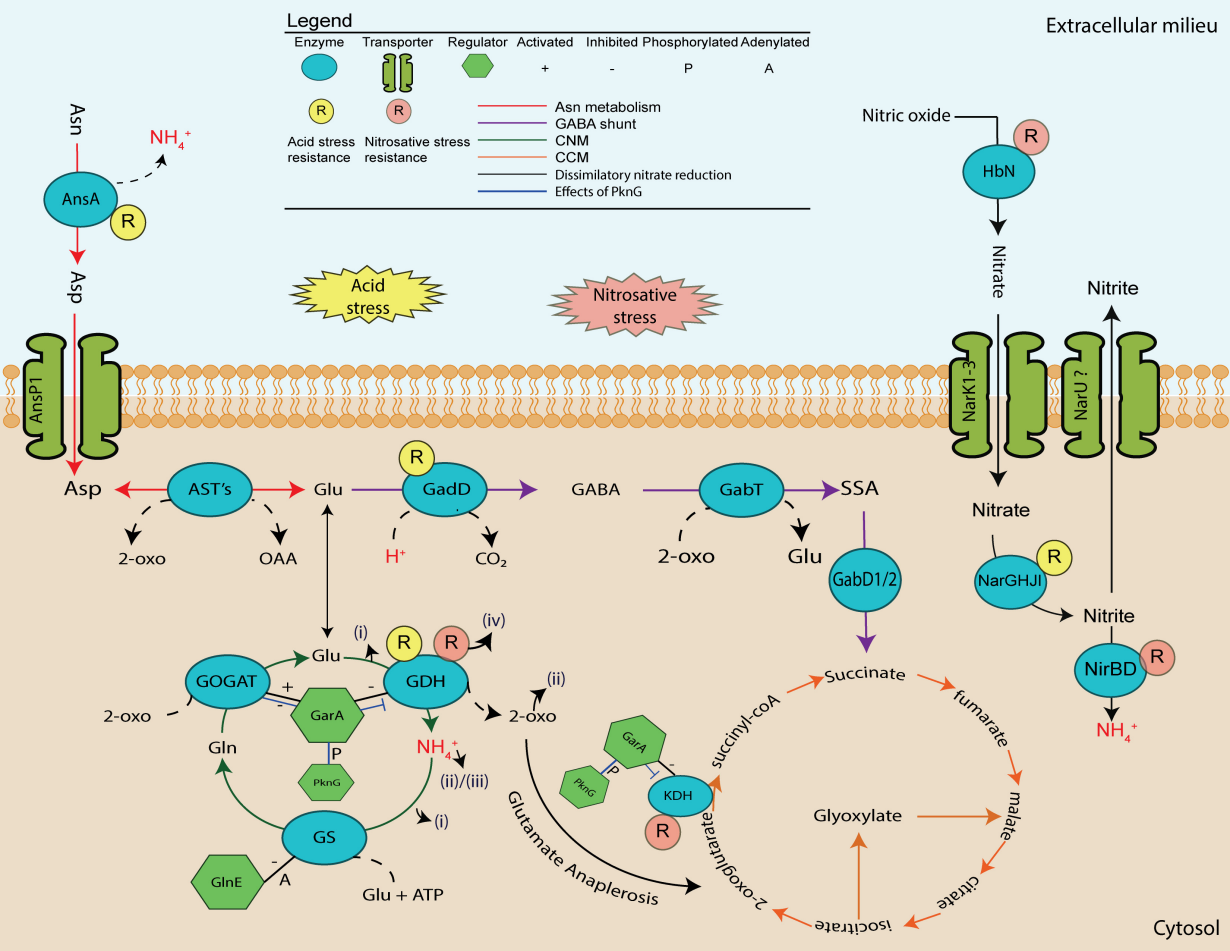
The role(s) of enzymes related to central mycobacterial glutamate metabolism and metabolites in the resistance against acid and nitrosative stress. Glutamates is an important nitrogen source (i), carbon source (ii) protectant against acidic stress (iii) as well as against nitrosative stress (iv). Metabolites shown in red are responsible for resistance against either nitrosative stress or acidic stress. Enzymes marked with circles have been demonstrated to be required for resistance against cellular stress.

Hence, we propose the following model, depicted in Fig 6, whereby GDH plays a critical role in survival of pathogenic mycobacteria inside macrophages: (i) as an effector of nitrogen and (ii) carbon assimilation by degrading glutamate to release ammonium and 2-oxoglutarate, respectively, (iii) by protecting against acidic stress through release of buffering ammonia from glutamate degradation, and (iv) by protecting against nitrosative stress through a yet unknown mechanism which may involve priming of oxidative stress mechanisms by sustaining cytosolic ammonia levels or through the release of the 2-oxoglutarate backbone of the glutamate molecule.

## Conclusion

Unexpectedly, our data support a much more important role for GDH than GOGAT in the intracellular lifestyle of slow growing mycobacteria, such as *M*. *tuberculosis*. The unique properties of L180 GDH compared to other characterised GDHs, including a very large subunit size, exclusive NAD^+^ co-enzyme specificity, and exclusive distribution among bacteria may have positive implications for the potential of L180 GDH as a specific anti-TB drug target [45]. A protein BLAST of *M*. *tuberculosis gdh* against the genomes for common intestinal bacterial genera, including *Bacteroides*, *Enterococcus*, *Escherichia*, *Klebsiella*, *Staphylococcus*, *Lactobacillus* and *Clostridium* delivered no homologues, which may further qualify L180 GDH as a specific anti-TB drug target (query coverage < 40 for only two Lactobacilli and one Bacteroides, http://blast.ncbi.nlm.nih.gov/Blast.cgi).

## Supporting Information

S1 Fig The percentage survival of wild type *M*. *bovis* BCG, *Δgdh* and *Δgdh* complement strains 48 hours post challenge with 500 μM DETE/NO. All strains were cultivated in excess ammonium sulphate (20 mM or 30 mM). Cells were washed 6 times with PBS prior to DETE/NO challenge and diluted to an OD_600_ 0.0005. Percentages were calculated from CFU enumerations. Data was obtained from three independent experiments.
(TIF)

S1 File Isolation of total RNA, cDNA and qRT-PCR methods. (PDF)

S2 File GDH activity assay methods. (PDF)

S1 Table Expression of GDH in wild type BCG, *Δgdh* mutant and *Δgdh* complement cultured in 7H9 to mid-logarithmic growth phase (OD_600_ = 0.5–0.7). (PDF)

S2 Table *Mycobacterium bovis* BCG strains used in this study. (PDF)
