# Supplementary material for: Glutamate Dehydrogenase Is Required by Mycobacterium bovis BCG for Resistance to Cellular Stress
Source: PLoS One. 2016 Jan 29;11(1):e0147706. doi: 10.1371/journal.pone.0147706 (PMC4732601; doi:10.1371/journal.pone.0147706)
Supplement: S1 File — (PDF) [file pone.0147706.s002.pdf]

Isolation of total RNA, cDNA conversion and qRT-PCR methods

Bacteria (15 ml) were pelleted by centrifugation, re-suspended in 1 ml RNApro solution (MP Biomedicals, Germany) and transferred to a Lysing Matrix B tube (MP Biomedicals, Germany). Bacterial cells were homogenised twice with a FastPrep-24 tissue and cell homogeniser (MP Biomedicals, Germany) for 40 seconds at a speed setting of 6.5 m/s (samples were cooled on ice for 2 – 4 minutes between homogenisations). The Fast RNA Pro Blue Kit protocol (MP Biomedicals, Germany) was subsequently followed to extract RNA up until the point when ethanol was added to precipitate nucleic acids. At this point, the ethanol-containing solution was transferred directly to the membrane of a NucleoSpin RNA II column (Macherey Nagel, Germany). RNA was subsequently purified according to the manufacturer's recommendations (Macherey Nagel, Germany), which included an on-column DNase-treatment step. RNA extraction, analysis and cDNA conversion was performed in duplicate for each assayed bacterial culture. RNA purity was analysed by spectrophotometric profiling on the NanoDrop 1000 spectrophotometer (Thermo Scientific, USA). Samples with A260/A280 and A260/A230 ratios between 1.80 and 2.20 were considered to be pure of contaminating reverse transcriptase or DNA polymerase inhibitors. Integrity of RNA was assessed as well as RNA concentration determined by automated electrophoresis with the Agilent 2100 Bioanalyzer (Agilent Technologies, USA). Samples with RIN values greater than 7.5 were considered undegraded and used in subsequent analyses. RNA (400 ng) was converted to cDNA using the PrimeScript 1st Strand cDNA Sunthesis kit (Takara, Japan) and random hexamer primers according to the manufacturer's recommendations. After synthesis a 1:2 dilution of the cDNA reaction was prepared for use in subsequent steps. For the gene expression analysis of the high copy number reference gene *rrs* (encodes for

16S ribosomal RNA), further 1:100 000 dilutions were made. This was done in order for the threshold cycle (Ct) values of assays with these genes to occur in the linear range of the PCR reaction (between 15 and 25 cycles). A no reverse transcriptase (-RT) control was included for each sample. Real time PCR was carried out in a 384-well plate format with the 7900HT real time instrument (Applied Biosystems, USA) using the TaKaRa SYBR Premix Ex Taq (Tli RNaseH Plus) kit (Takara, Japan), according to the manufacturer's recommendations. Each reaction (10 µl) contained 10 ng cDNA template and was performed in duplicate. In order to control for gDNA contamination of cDNA samples –RT controls (10 ng nucleic acid) as wells as Turbo DNase-treated RNA (100 ng) were assayed in parallel with sample assays. The assay for the reference gene, *rrs*, was used in the qPCR reactions of RNA and –RT controls. A threshold cycle (Ct) value of lower than 32 for a –RT control resulted in exclusion of a sample from further analysis. A standard curve of Ct over target template number was obtained for each assay from a ten-fold dilution series of purified wt-BCG gDNA ranging from 10^6 genomes/qPCR reaction to 10 genomes/qPCR reaction. Absolute expression was determined from the linear range of the standard curve obtained for each assay. Absolute gene expression data for gene of interest was normalised to that of the reference gene *rrs*. *rrs*-normalised data of treatment groups were compared to *rrs*-normalised data of untreated groups to obtain a measure of relative gene expression in response to treatment and analysed statistically (as indicated in the text).
