# Supplementary material for: Glutamate Dehydrogenase Is Required by Mycobacterium bovis BCG for Resistance to Cellular Stress
Source: PLoS One. 2016 Jan 29;11(1):e0147706. doi: 10.1371/journal.pone.0147706 (PMC4732601; doi:10.1371/journal.pone.0147706)
Supplement: S2 File — (PDF) [file pone.0147706.s003.pdf]

GDH activity assay methods

M. bovis BCG (25 ml) was pelleted (2000 × g, 10 minutes, 4°C), re-suspended in 1 ml of 100mM NaH2PO4/K2HPO4 buffer (pH 7.0), transferred to a 2 ml screw cap tube containing approximately 200 µl zirconium balls (0.1 mm diameter), homogenised with a FastPrep-24 tissue and cell homogeniser (MP Biomedicals, USA) for 40 seconds at a speed setting of 6.5 m/s, and cooled on ice for 2 – 4 minutes. Homogenisation was repeated once more with the same settings. The homogenate was centrifuged (12 000 × g, 10 minutes, 4°C) and the clear cell lysate transferred to a sterile 1.5 ml microcentrifuge tube. Total protein concentration in lysates was determined by method of the Bio-Rad Protein Assay (Bio-Rad, USA) and the manufacturer's standard protocol for a microtiter plate. GDH activity was assayed at 37°C in the forward or reverse direction in a 96 well plate format according to the method of Miñambres et al. (2000) [15]. A single forward reaction mixture (200 µl, pH = 7) contained 100 mM NaH2PO4/K2HPO4, 100 mM NH4Cl, 15 mM 2-oxoglutarate, 0.15 mM NADH and 10 - 20 µg total protein. A single reverse reaction mixture (200 µl) contained 100 mM NaH2PO4/K2HPO4, 100 mM L glutamate, 0.5 mM NAD+ and 10 - 20 µg total protein. Reactions were initiated with the addition of the cofactor NADH (forward reaction) or NAD+ (reverse reaction). The continuous enzyme activity assay tracks the oxidation of NADH to NAD+ over 1-minute intervals for a 10 minute period by measuring a decrease in optical density at 340nm. Spectrophotometric measurements were made in a 96 well plate format with a Synergy HT Luminometer (Bio-Tek Instruments, USA). As the path length of the light through the enzyme reaction is not fixed at 1cm in the microtiter plate assay the molar extinction coefficient of NADH, expressed in units of OD340nm.µmol 1.well 1, was determined empirically using a standard curve of OD340nm over [NADH]. For each sample a control reaction was included in which 2-

oxoglutarate was omitted. One unit (U) of GDH activity was defined as the amount of enzyme which consumed 1 nanomole of NADH per minute. Specifc GDH activity was expressed as mU per mg of total protein (mU/mg protein).
