# Supplementary material for: Glutamate Dehydrogenase Is Required by Mycobacterium bovis BCG for Resistance to Cellular Stress
Source: PLoS One. 2016 Jan 29;11(1):e0147706. doi: 10.1371/journal.pone.0147706 (PMC4732601; doi:10.1371/journal.pone.0147706)
Supplement: S1 Table — (PDF) [file pone.0147706.s004.pdf]

Table S1: Expression of GDH in wt-BCG, *Δgdh* mutant and *Δgdh* complement cultured in 7H9 to mid-logarithmic growth-phase (OD_{600} = 0.5-0.7).

**Strain**	**Expression**	
**<u>mU/mg protein</u>**		
wt-BCG	40.82 ± 6.26	
Δgdh	0.39 ± 0.40	***
Δgdh complement	1.06 ± 0.53	***
**<u>16S-normalized expression of *gdh*</u>**		
wt-BCG	2.95 ± 0.72	
Δgdh	0.00 ± 0.00	[na]
Δgdh complement	0.50 ± 0.09	*

All procedures followed for GDH specific activity determination and quantification of relative *gdh* mRNA levels are described in the supplementary materials and methods file.

Mean specific activity (mU/mg protein) ± standard deviation was calculated from triplicate culture data.

Mean specific activity was compared between the *Δgdh* mutant or *Δgdh* complement and wt-BCG and differences were analysed by one way ANOVA with Bonferroni post-testing.

⋆⋆⋆ - significantly different from wt-BCG $p < 0.001$

Mean 16S-normalized expression of *gdh* ± standard error was calculated from three independent experiments carried out in duplicate.

Mean 16S-normalized expression of *gdh* was compared between the *Δgdh* complement strain and wt-BCG and the difference analysed by unpaired two-tailed t-test with Welch's correction.

⋆ - significantly different from wt-BCG $p < 0.05$, na – not applicable
