# Supplementary material for: Glutamate Dehydrogenase Is Required by Mycobacterium bovis BCG for Resistance to Cellular Stress
Source: PLoS One. 2016 Jan 29;11(1):e0147706. doi: 10.1371/journal.pone.0147706 (PMC4732601; doi:10.1371/journal.pone.0147706)
Supplement: S2 Table — (PDF) [file pone.0147706.s005.pdf]

Table S2. *Mycobacterium bovis* BCG strains used in this study.

Strain:	Description:	Source/reference:
wt-BCG	*M. bovis BCG* str. *Pasteur 1743P2*	Laboratory collection
ΔgltBD	wt-BCG pJV53 background in which the *gltBD* operon was substituted with a hygromycin cassette, hyg^R, kan^R	[1]
ΔgltBD complement	Genetically complemented strain of *ΔgltBD*, *attB*::pGCgltBD, carries pJV53, hyg^R, kan^R, $gent^R$	[1]
Δgdh	wt-BCG background in which the *NruI* fragment spanning the GDH domain within *gdh* was deleted.	[1]
Δgdh complement	Genetically complemented strain of *Δgdh*, *attB*::pGCgdh, $gent^R$	[1]

1. Viljoen AJ, Kirsten CJ, Baker B, van Helden PD, Wiid IJF. The Role of Glutamine Oxoglutarate Aminotransferase and Glutamate Dehydrogenase in Nitrogen Metabolism in Mycobacterium bovis BCG. PLoS ONE. 2013;8. doi:10.1371/journal.pone.0084452
